# Forecasting of landslide displacements using a chaos theory based wavelet analysis-Volterra filter model

**DOI:** 10.1038/s41598-019-56405-y

**Published:** 2019-12-27

**Authors:** Yuanyao Li, Ronglin Sun, Kunlong Yin, Yong Xu, Bo Chai, Lili Xiao

**Affiliations:** 1 Institute of Geological survey, China University of Geosciences, Wuhan, 430074 China; 2 School of Environmental Studies, China University of Geosciences, Wuhan, 430074 China; 3 Wuhan Center, China Geological Survey, Wuhan, 430205 China; 4 School of Highway, Chang’an University, Xi’an, 710064 China

**Keywords:** Environmental impact, Natural hazards

## Abstract

Landslide displacement time series can directly reflects landslide deformation and stability characteristics. Hence, forecasting of the non-linear and non-stationary displacement time series is necessary and significant for early warning of landslide failure. Traditionally, conventional machine learning methods are adopted as forecasting models, these forecasting models mainly determine the input and output variables experientially and does not address the non-stationary characteristics of displacement time series. However, it is difficult for these conventional machine learning methods to obtain appropriate input-output variables, to determine appropriate model parameters and to acquire satisfied prediction performance. To deal with these drawbacks, this study proposes the wavelet analysis (WA) to decompose the displacement time series into low- and high-frequency components to address the non-stationary characteristics; then proposes thee chaos theory to obtain appropriate input-output variables of forecasting models, and finally proposes Volterra filter model to construct the forecasting model. The GPS monitoring cumulative displacement time series, recorded on the Shuping and Baijiabao landslides, distance measuring equipment monitoring displacements on the Xintan landslide in Three Gorges Reservoir area of China, are used as test data of the proposed chaotic WA-Volterra model. The chaotic WA-support vector machine (SVM) model and single chaotic Volterra model without WA method, are used as comparisons. The results show that there are chaos characteristics in the GPS monitoring displacement time series, the non-stationary characteristics of landslide displacements are captured well by the WA method, and the model input-output variables are selected suitably using chaos theory. Furthermore, the chaotic WA-Volterra model has higher prediction accuracy than the chaotic WA-SVM and single chaotic Volterra models.

## Introduction

The safety of local people’ life and property are threatened seriously by the reservoir landslides distributing along the Three Gorges Reservoir^1–3^. Forecasting of Landslide displacements is considered as an important part of an operational early warning system^2,4–7^. Hence, it is crucial and significant to forecast the landslide displacements for early warning^8^.

Over the past decades, Global Position System (GPS) has been widely and successfully used to monitor the landslide displacement time series^9^. GPS technology makes it possible to real-timely track the deformation processes of a landslide. The landslide displacement prediction models mainly include physically based models and data-based models^6,10^. The data-based models are established through training and testing the input-output variables using linear and/or non-linear models. The modeling processes of data-based models are more simple and accurate than those of physically based models^6,11^. Hence, this study propose data-based models to forecast the landslide displacement time series.

Some data-based models namely the classical black box models (Auto Regressive, Moving Average, Multiple Linear Regression, *et al*.) have been proposed for time series prediction since 1970^12^. These classical black box models are not accurate enough for nonlinear time series prediction because they are linear models. In the past three decades, many Artificial Intelligence (AI) methods, which provide a way to solve the problems of complexity, dynamism and nonlinear characteristics in nonlinear time series, had been used to predict landslide displacement time series. These models include artificial neural networks (ANNs)^13–16^, extreme learning machine^17^, fuzzy logic approach^18^ and support vector machine (SVM)^19–22^, *et al*.

However, the nonlinear time series prediction performances of these AI methods are limited. This is because that, (1) the nonlinear and non-stationary characteristics of landslide displacement time series are not captured fully by single AI models; (2) the input-output variables of the AI methods are determined empirically without appropriate determination law for input-outputs; and (3) the nonlinear fitting and predicting abilities of these AI models should be improved effectively^4,11,19,23,24^. In additional, related literature indicates that AI models have some disadvantages of local optimum, slow training and testing rate and over-fitting problem, which also hinder the prediction accuracy for the nonlinear time series^2,4^. To address these three limitations existed in AI models, this study proposes a chaos theory based wavelet analysis-Volterra filter model (chaotic WA-Volterra model) for displacements prediction.

For the first limitation, wavelet analysis (WA) is used to address the serious nonlinear and non-stationary problems in the landslide displacement. WA is an effective signal processing tool that can solve the nonlinear and non-stationary signal and offer the time-scale localization for signals^25^. The main features of WA method are exploring the time series from both time domain and frequency domain, which provide meaningful information for the physical structure of time series^15^. Hence, WA based AI models firstly decompose a time series into several multi-resolution frequency components and then these components are respectively predicted in the AI models with higher prediction performance^13,14^. Recently, WA-AI models have been effectively introduced to the areas of reservoir fluid contacts prediction^26^, stream flow data series prediction^14^ and seasonal variation of landslide displacement^27^. This study also introduces WA into AI methods for non-linear and non-stationary cumulative landslide displacements prediction.

In addition, the prediction performance of AI method is essentially depend on the input-output variables. In this study, the chaos theory, which can track the evaluation of nonlinear time series and rebuild its original evaluation system, is used to determine the input-output variables of the AI models. Chaos theory is an important method for input-output variables selection which is widely used in many areas^6,28^. In theory, based on the determination of the chaos evidence of landslide displacements, embedding theory and phase space reconstruction (*PSR*) methods of chaos theory can be used to build a chaos theory based model for displacements prediction. Based on embedding theory^29^, the landslide displacements might to be predicted using a single variable displacement time series. Meanwhile, on the basis of *PSR* method^30^, a single variable displacement time series can be constructed into a multi-dimensional phase-space to obtain the input-output variables of prediction models.

Furthermore, after the conductions of chaos theory and WA method on landslide displacements, the Volterra filter model is innovatively introduced into this study^31^. The Volterra filter model can predict the chaotic time series using a few time series data in the training and testing processes, and can automatically track the motion trace of the chaotic time series. Hence, the Volterra filter model can overcome the disadvantages of traditional machine learning models, and it has been successfully used in many areas with excellent generalization performance, such as air/fuel ratio prediction^32^, multi-scale stream flow forecasting^33^ and traffic flow prediction^34^. However, no attention has been attracted to use the Volterra filter model to predict landslide displacements, although the Volterra filter model has efficient prediction performance.

To summarize, a chaotic WA-Volterra model is innovatively proposed in this study to overcome the drawbacks of traditional machine learning models. Meanwhile, the chaotic WA-SVM model and the single chaotic Volterra filter model without WA method are used for comparisons. The GPS monitoring monthly displacement time series of the Shuping landslide and Baijiabao landslide, distance measuring equipment monitoring displacements on the Xintan landslide in Three Gorges Reservoir area (TGRA) are used as case study.

## Study Area and Materials

**Figure 1:**
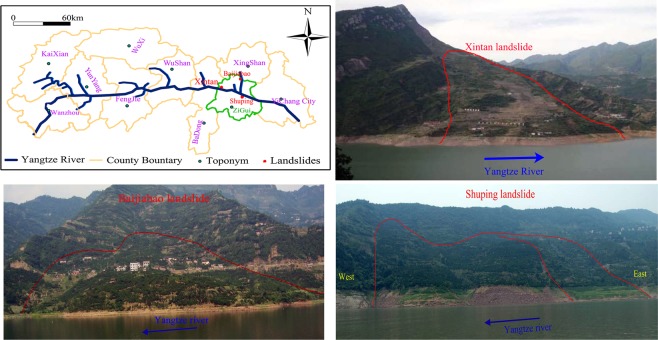
Geographic location information of Shuping and Baijiabao landslides (Adobe Photoshop CS3, http://www.adobe.com/cn/products/photoshop.html, drawn by Yuanyao Li; the photograph is taken from Yuanyao Li).

The locations of the Shuping, Baijiabao and Xintan landslides are shown in Fig. 1; Shuping landslide locates on the south side of the Yangtze river in ZiGui County. The Baijiabao landslide is located on the west side of Xiangxi river, a tributary of the Yangtze river. Xintan landslide locates on the north side of the Yangtze river in ZiGui County.

### Geological conditions and deformation characteristics of the shuping landslide

**Figure 2:**
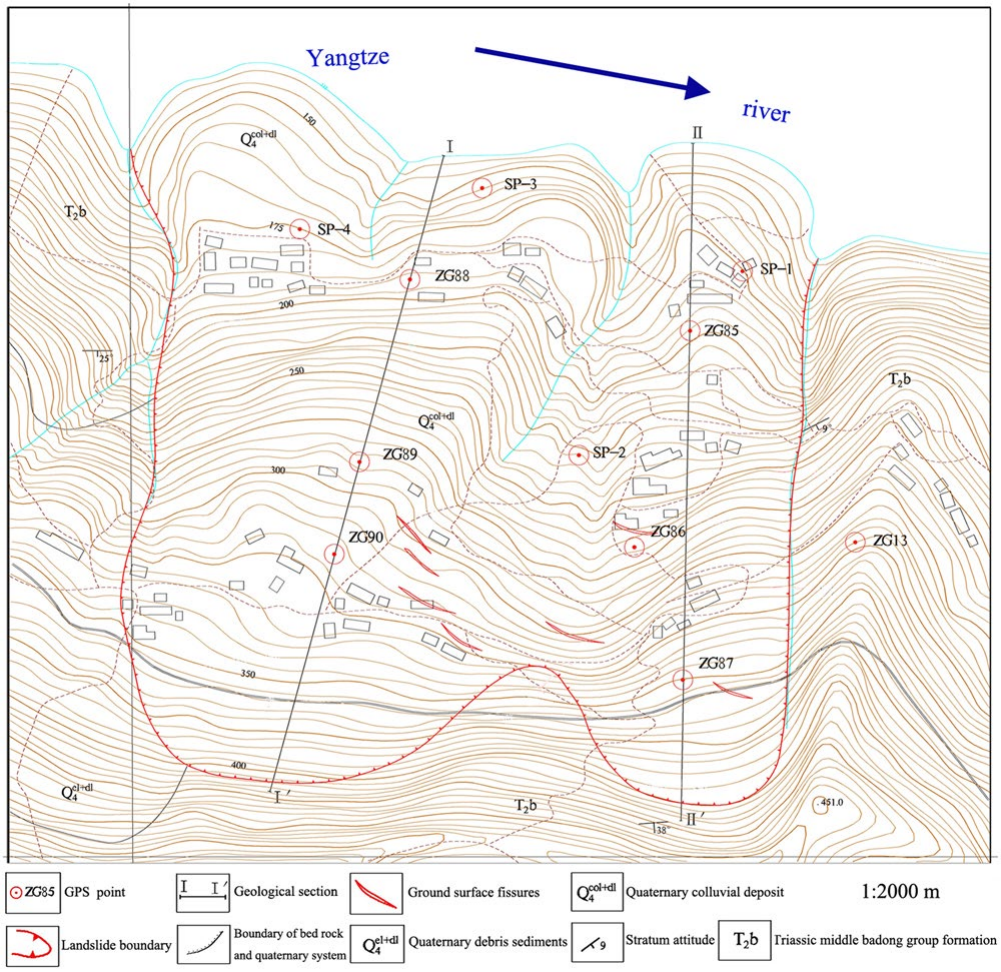
Topographical map of Shuping landslide, with locations of GPS points (AUTOCAD 2014, https://www.autodesk.com.cn/, drawn by Yuanyao Li).

The topographic map of the research site and its GPS monitoring network are shown in Fig. 2. Figure 2 shows that the upper boundary of the chair-like shaped Shuping landslide is defined by the bedrock-soil interface. The maximum elevation of the upper boundary is 415 m. Its toe elevation is approximately 144 m. The longitudinal dimension and width of the landslide are 800 m and 700 m, respectively. In addition, the mean depth of the sliding surface is approximately 55 m.

The materials of the landslide are composed of medium permeable quaternary deposits. The slope mass structure is loose and it varies from 10° to 35°. The dip direction and dip angle are approximately 143° and 15°, respectively. The slip zone is composed of silty clay and fragmented rubble. The bedrock lithology is characterized by triassic formation of middle Badong formation.

**Figure 3:**
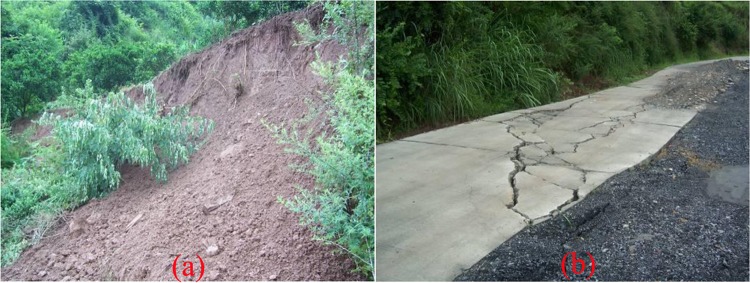
Deformation characteristics of Shuping landslide (photographs taken by Yuanyao Li).

Shuping landslide was activated in June 2003 and it exhibited a large amount of local deformation and failure. Some reservoir bank collapses occurred in the frontal part of this landslide from October 2003 to January 2004. Surface investigations showed that shearing and crush-pressing cracks had occurred since January 2004. The deformation of the study area had rapidly progressed since April 2007. Figure 3(a) illustrates a small debris flow that occurred. Figure 3(b) shows a large amount of significant road deformation and cracking that occurred in the road at the upper part of the landslide. The deformation and failure characteristics of Shuping landslide are serious.

In general, the deformation and failure of reservoir landslides are related to the rapid changes in the groundwater seepage field^35–37^. The mechanical effects of hydraulic uplift pressure and reverse seepage pressure on the landslide will increase when the groundwater level rises^38^. Meanwhile, the physical effects of groundwater seepage on landslides are mainly reflected in the reduction of soil shear strength and in the softening of sliding surface^39,40^. However, it is not easy to obtain sufficient groundwater level time series for building prediction models. Hence, this study does not put groundwater levels into the chaotic WA-Volterra model for landslide displacements prediction.

### Geology and deformation characteristics of baijiabao landslide

**Figure 4:**
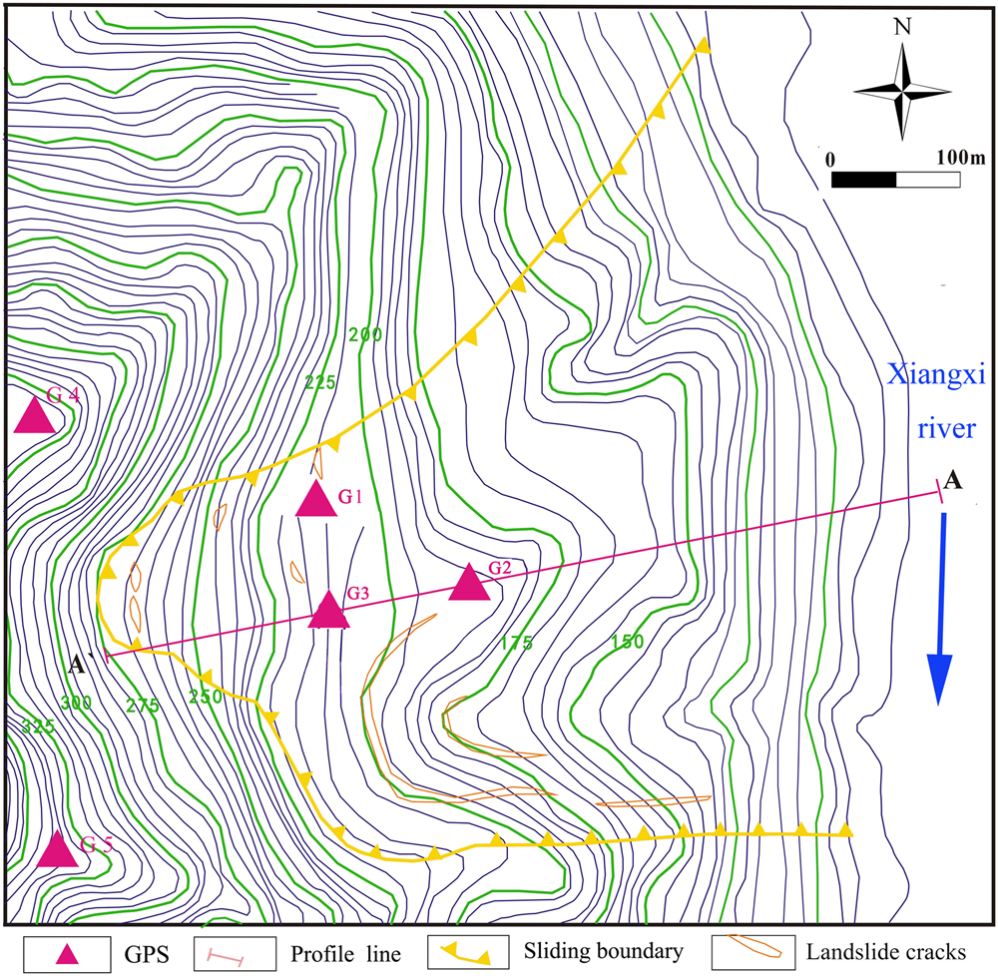
Terrain map of the Baijiabao landslide with GPS locations (AUTOCAD 2014, https://www.autodesk.com.cn/, drawn by Yuanyao Li).

Baijiabao landslide (Fig. 4), which is a fan shape and destructive landslide, is composed by the quaternary deposits (silty clay interspersed with fragmented stone). The structure of these quaternary deposits is loose and porous. The bedrock under this sliding mass is composed by Jurassic mudstone and sandstone. In addition, the Baijiabao landslide is a large landslide with an approximately volume of 9.9 × 10^6^ m^3^, an approximately area of 2.2 × 10^5^ m^2^ and an approximately sliding depth of 45 m. The average slope of Baijiabao landslide is about 12°, the elevation of the frontal part of the landslide is about 125 m (extending to the bed of Xiangxi River) and the elevation of the upper part is about 275 m. Figure 4 shows that the boundaries of Baijiabao landslide are identified as gully and bed rock.

### Geology and deformation characteristics of baijiabao landslide

The Xintan landslide (Fig. 1) is an accumulate landslide composed by silty clay and sandstone fragments. Its elevation ranges between 70 m and 500 m from frontal part to the upper part of the landslide with thickness of accumulative layer about 25.5 m. The Xintan landslide is generally inclined towards to the Yangtze river with average slope of 25° and a multilevel terraced terrain. The right and left landslide boundaries are characterized by steep cliffs. The bedrock lithology of Xintan landslide is mainly featured as Silurian sandstone and shale. Its groundwater is replenished by rainfall. There is almost no groundwater in this landslide because the slide mass is loose and the discharge of the groundwater is very good.

There are several reasons for the landslide instability. One reason is that, the surface slope of this landslide is steep, the accumulative layer of this landslide is loose and there is weak intercalated layer in this landslide. Another reason is that, the seasonal heavy rainfall decreases the shear strength of this landslide, as a result, the landslide is reactivated. The deformation and instability processes of Xintan landslide began in January 1977, and completely destroyed in May 1985. The local people’s life and properties are seriously threatened by the deformation of Xintan landslide.

### GPS system building on shuping and baijiabao landslides

To monitor the displacement time series of the Shuping and Baijiabao landslides, the GPS system was respectively built on the Shuping landslide in June 2003 and built on the Baijiabao landslide in September 2006. Figure 2 shows that the GPS points ZG85 ~ ZG90 were set as observation points, while the points ZG83 and ZG84 were set as reference points. Two GPS reference points were placed in the stable zones outside the Shuping landslide to ensure that there were no deformations in the reference points. Six GPS observation points were built in the deformation zone of the landslide. The same as Shuping landslide, Baijiabao landslide was also monitored by the GPS system with G4 ~ G5 as reference points and G1 ~ G3 as observation points (Fig. 4).

**Figure 5:**
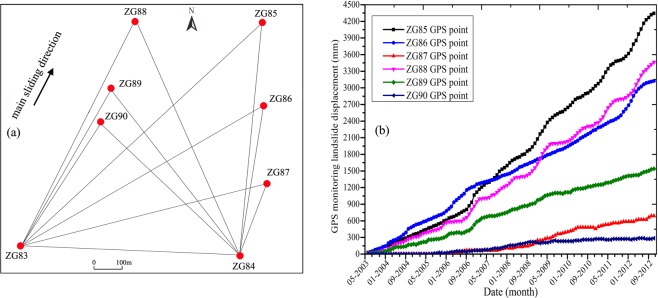
GPS system on Shuping landslide and obtained cumulative displacements with ZG83 ~ ZG90 GPS points (Drawn by Yuanyao Li).

**Figure 6:**
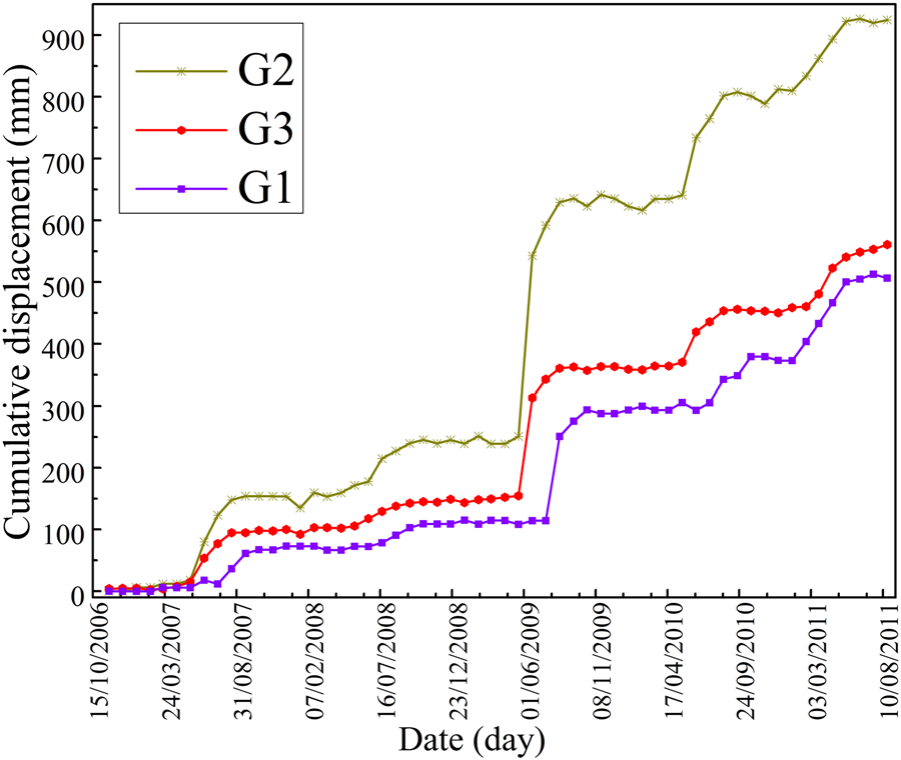
GPS monitoring cumulative displacements of Baijiabao landslide with G1 ~ G3 GPS points (Drawn by Yuanyao Li).

The reference stations respectively constructed a landslide monitoring control network with each GPS observation points ZG85 ~ ZG90 (Fig. 5(a)) and G1 ~ G3 in turn every month. Then, the displacements of GPS observation points are calculated comparing to the reference points based on the constructed landslide monitoring control network. The GPS receiving signals were processed by baseline processing and network adjustment in the GAMIT/GLOBK software^41^. Then, the monitored cumulative displacements can be obtained and displayed. In this study, two series of GPS monitoring landslide displacement time series were obtained from June 2003 to November 2012 on Shuping landslide (Fig. 5(b)) and from October 2006 to August 2011 on Baijiabao landslide (Fig. 6).

#### GPS monitoring displacements of shuping landslide

Figure 5(b) indicates that the cumulative displacements of the Shuping landslide are of non-linear and non-stationary characteristics. Figure 5(b) also shows that the cumulative displacement values of points ZG85, ZG86 and ZG88 on the frontal part of the landslide are greater than those of the points on the upper part, which suggests that the Shuping landslide is a translational landslide with earth sliding^42^. In this study, the cumulative displacements of ZG85 ~ ZG88 points with non-linear and non-stationary characteristics are predicted using the proposed models.

#### GPS monitoring displacements of baijiabao landslide

The Baijiabao landslide has been activated by the water level fluctuation of Three Gorges Reservoir and the seasonal rainfall since September, 2006. As a result, some shear and tensile fissures occurred in the middle and upper parts of this landslide (Fig. 4). Three GPS sensors have been placed on the landslide for landslide displacements monitoring since September, 2006 (Fig. 6). The nonlinear and non-stationary deformation features of Baijiabao landslide are reflected by these three displacement curves. Furthermore, in order to verify the prediction performance of WA-Volterra model for landslide displacement prediction, the G3 GPS point is used in this study as study case.

### Creep deformation characteristics of the three landslides

**Figure 7:**
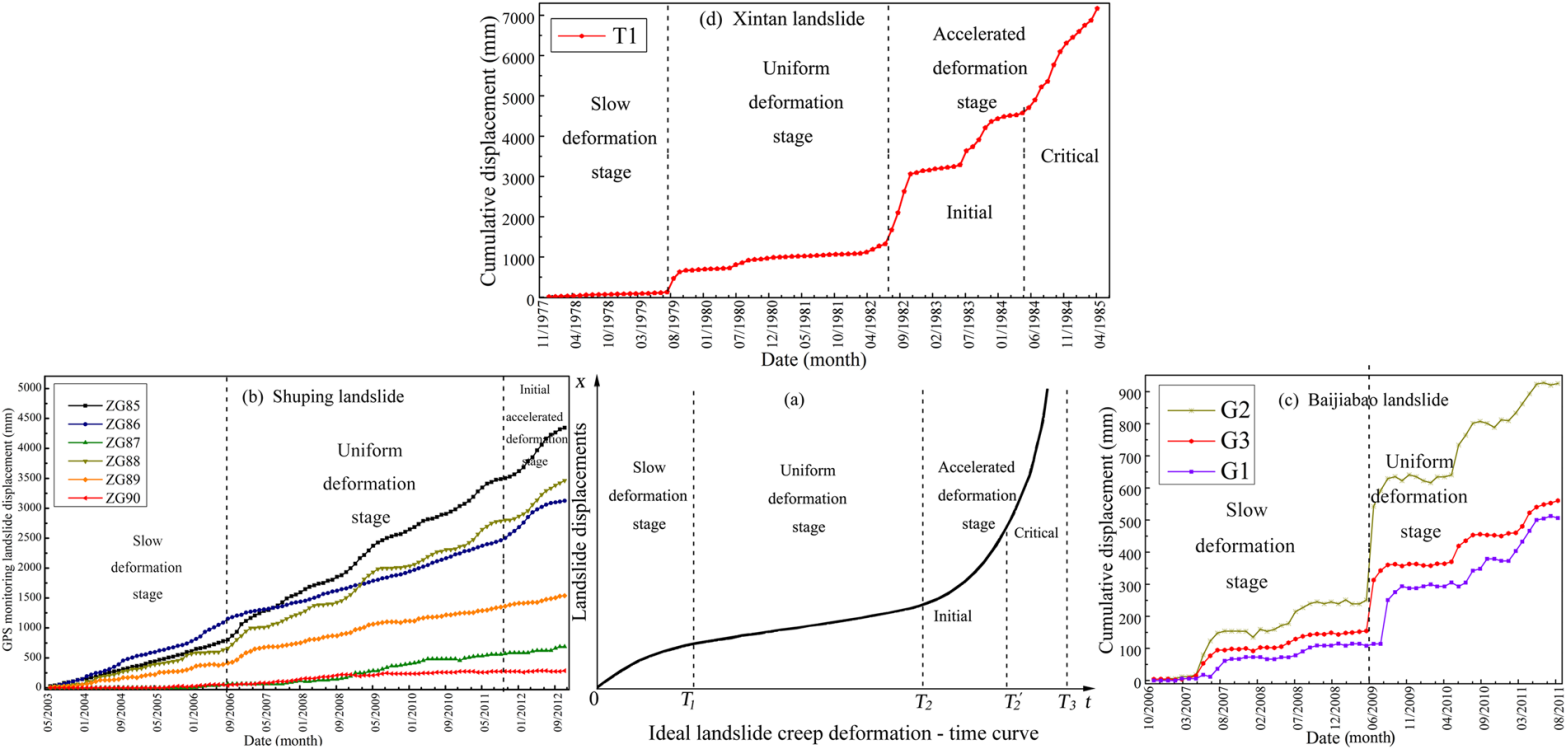
landslide ideal creep deformation-time curve (**a**), and deformation characteristics analysis of Shuping landslide (**b**) and Baijiabao landslide (**c**).

Related literature shows that, the nature of soil landslide failure can be regarded as the creep deformation process of sliding mass^43,44^. In general, according to the landslide deformation rate and cumulative displacements, the ideal landslide creep deformation-time curve can be divided into three stages^44^: slow deformation stage with small deformation rate and low slope of cumulative displacements (0 ~ *T*_1_), uniform deformation stage with almost uniform deformation rate and gradually increasing cumulative displacements (*T*_1_ ~ *T*_2_), and accelerated deformation stage with high deformation rate and rapidly increasing cumulative displacements until landslide failure occurs (*T*_2_ ~ *T*_3_) (Fig. 7(a)). The last stage can be further divided into initial accelerated deformation and critical failure stages.

The landslide deformation falling into the accelerated deformation stage is a very important warning indicator of landslide failure^45^. Therefore, it is significant to analysis the deformation features according to the creep deformation-time curves. Specially, The cumulative displacements of Shuping and Baijiabao landslides belong to non-ideal creep deformation-time curves. Instead, the cumulative displacements of Xintan landslide belongs to ideal creep deformation-time curve. The deformation of Shuping landslide is a type of continuous and almost uniform creep deformation feature, with slow deformation stage from June 2003 to September 2006, uniform deformation stage from October 2006 to October 2011 and initial accelerated deformation stage from November 2011 to January 2013 (Fig. 7(b)). Meanwhile, the deformation of Baijiabao landslide has a type of seasonal step-like creep deformation feature, with slow deformation stage from October 2006 to June 2009 and uniform deformation stage from July 2009 to August 2011(Fig. 7(c)). In addition, the Xintan landslide has also a seasonal step-like creep deformation feature, with slow deformation stage from January 1978 to August 1979, with uniform deformation stage from September 1979 to July 1982, and then with critical accelerated deformation stage from May 1984 until this landslide was completely destroyed(Fig. 7(d)).

## Results

### Chaos evidences identification of monthly cumulative displacements

Related literature shows that the chaos theory based models have been widely studied in many areas of non-linear time series prediction, such as water level prediction^35^ and electronic power prediction^46^. In this study, the phase spaces of GPS monitoring displacement time series are reconstructed firstly, then their chaos evidences are identified using the LLE and *CD* methods.

#### Reconstruct the phase spaces of cumulative displacements

The phase spaces of ZG85 (*x*_*i*_^*ZG*85^) ~ ZG88 (*x*_*i*_^*ZG*88^)(*i* = 1, 2, ……, 114) of Shuping landslide, G3 (*x*_*i*_^*G*3^) (*i* = 1, 2, ……, 57)of Baijiabao landslide, T1 (*x*_*i*_^*T*1^) (*i* = 1, 2, ……, 89)of Baijiabao landslide, cumulative displacement time series are reconstructed. The *τ* of ZG85 ~ ZG88, G3 and T1 displacements are set to 1 and the optimal *m* values of ZG85 ~ ZG88, G3 and T1 displacements are respectively 3, 2, 3, 3, 5 and 3 based on the false nearest neighbor method. These reconstructed phase spaces are shown in Table 1.

**Table 1:** Reconstructed phase spaces of ZG85 ~ ZG88, G3 and T1 GPS monitoring cumulative displacements.

Displacements	Reconstructed phase spaces (input variables)	Output variables
ZG85	${X}_{i}^{ZG85}=({x}_{i}^{ZG85},{x}_{(i-1)}^{ZG85},\,{x}_{(i-2)}^{ZG85}),i=3,4,\cdots ,113$	${x}_{(i+1)}^{ZG85}$
ZG86	${X}_{i}^{ZG86}=({x}_{i}^{ZG86},{x}_{(i-1)}^{ZG86}),i=2,3,\cdots ,113$	${x}_{(i+1)}^{ZG86}$
ZG87	${X}_{i}^{ZG87}=({x}_{i}^{ZG87},{x}_{(i-1)}^{ZG87},{x}_{(i-2)}^{ZG87}),i=3,4,\cdots ,113$	${x}_{(i+1)}^{ZG87}$
ZG88	${X}_{i}^{ZG88}=({x}_{i}^{ZG88},{x}_{(i-1)}^{ZG88},{x}_{(i-2)}^{ZG88}),i=3,4,\cdots ,113$	${x}_{(i+1)}^{ZG88}$
G3	${X}_{i}^{G3}=({x}_{i}^{G3},{x}_{(i-1)}^{G3},{x}_{(i-2)}^{G3}\,,{x}_{(i-3)}^{G3},{x}_{(i-4)}^{G3}),i=5,6,\cdots ,56$	${x}_{(i+1)}^{G3}$
T1	${X}_{i}^{T1}=({x}_{i}^{T1},{x}_{(i-1)}^{T1},{x}_{(i-2)}^{T1}\,),i=3,4,\cdots ,88$	${x}_{(i+1)}^{T1}$

#### Chaos evidence of monthly cumulative displacements

Final calculated *LLE* values of the ZG85 ~ ZG88, G3 and T1 displacement time series are respectively 0.0862, 0.0315, 0.0227, 0.0577, 0.1232 and 0.0526. The results show that there are chaos characteristics in all of the ZG85 ~ ZG88, G3 and T1 displacements because their *LLE* values are all greater than zero^47^. In addition, the non-linearity of the displacement time series can be measured by comparing the *LLE* values of the displacements. A greater *LLE* value means a higher non-linearity. The comparison results show that the G3 displacement has the highest non-linearity, while the ZG87 displacement has the lowest non-linearity.

**Figure 8:**
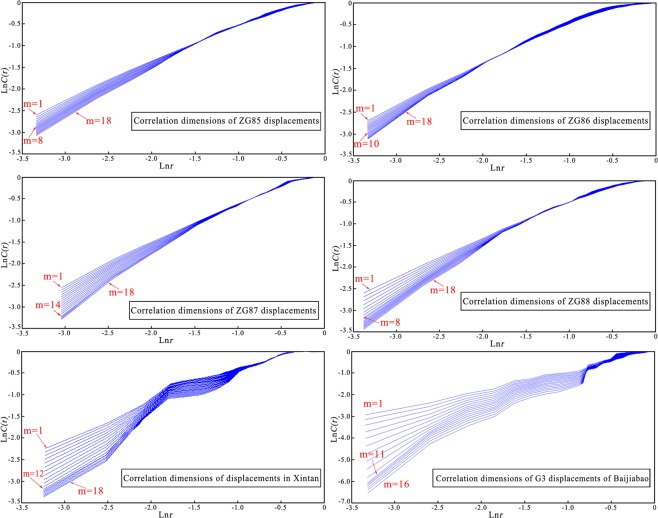
The correlation dimension curves of ZG85 ~ ZG88 of Shuping landslide, G3 of Baijiabao landslide and T1 of Xintan landslide cumulative displacements (drawn by Yuanyao Li).

*CD* method is also in this study, the corresponding *D*(*m*) are all calculated with the *m* increases from 1 to 18. It can be seen from Fig. 8 the relationships between *C*(*r*) and *r* of ZG85 ~ ZG88, G3 and T1 GPS monitoring displacements are respectively calculated. The calculation results indicate that the slopes of the lines in Fig. 8 respectively converge to a constant when *m* is increased to 8, 10, 14, 8, 11 and 12. Hence, we can draw conclusions that there are chaos characteristics in the ZG85 ~ ZG88, G3 and T1 GPS monitoring displacements, and the chaotic WA-Volterra and chaotic WA- SVM models can be adopted to predict the landslide displacements.

### Wavelet analysis of GPS monitoring landslide cumulative displacements

**Figure 9:**
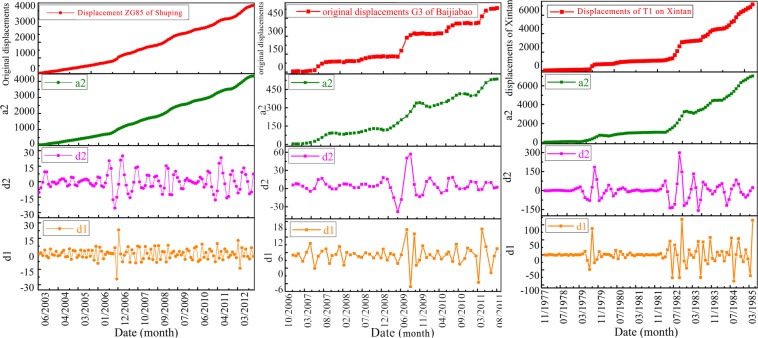
Displacement time series decomposition of ZG85 on Shuping landslide, G3 on Baijiabao landslide and T1 on Xintan landslide, with a2 reflecting low-frequency component, d1 reflecting the first high-frequency component and d2 reflecting the second high-frequency component of these cumulative displacements. (drawn by Yuanyao Li).

The chaotic WA-Volterra and WA-SVM models predict cumulative displacements through forecasting all the low- and high-frequency components. These components are obtained from the wavelet analysis of GPS monitoring cumulative displacements. The decomposition levels of ZG85 ~ ZG88 of Shuping landslide, G3 of Baijiabao landslide and T1 point of Xintan landslide cumulative displacements are all two according to Eq. (11). Then we can decompose the ZG85 ~ ZG88, G3 and T1 landslide cumulative displacements using WA method as:

1 $${x}_{i}^{ZG}={x}_{a2,\,i}^{ZG}+{x}_{d1,\,i}^{ZG}+{x}_{d2,i}^{ZG}$$

2 $${x}_{i}^{G}={x}_{a2,\,i}^{G}+{x}_{d1,\,i}^{G}+{x}_{d2,\,i}^{G}\,{\rm{and}}\,{x}_{i}^{T}={x}_{a2,\,i}^{T}+{x}_{d1,\,i}^{T}+{x}_{d2,\,i}^{T}$$

where *x*_*i*_^*ZG*^, *x*_*i*_^*G*^ and *x*_*i*_^*T*^ respectively presents the cumulative displacements of ZG85 ~ ZG88, G3 and T1; *x*_*a*2,*i*_, *x*_*d*1,*i*_ and *x*_*d*2,*i*_ respectively donates the low-frequency, the first high-frequency and the second high-frequency components of ZG85 ~ ZG88, G3 and T1 landslide cumulative displacements. Taking the ZG85, G3 and T1 cumulative displacements as examples, the final decomposition results of ZG85, G3 and T1 cumulative displacements are respectively shown in Fig. 9.

### Phase space reconstructions of each frequency component

The reconstructed phase spaces of each frequency component provide input and output variables of Volterra filter and SVM models. In this study, we set the *τ* of all the frequency components as one, and we calculate the optimal *m* of all the frequency components using the FNN method. The results show that the optimal *m* of *x*_*a*2,*i*_^*ZG*85^, *x*_*d*1,*i*_^*ZG*85^, *x*_*d*2,*i*_^*ZG*85^ are respectively 2, 4 and 4; *m* of *x*_*a*2,*i*_^*ZG*86^, *x*_*d*1,*i*_^*ZG*86^, *x*_*d*2,*i*_^*ZG*86^ are respectively 2, 5 and 3; *m* of *x*_*a*2,*i*_^*ZG*87^, *x*_*d*1,*i*_^*ZG*87^, *x*_*d*2,*i*_^*ZG*87^ are respectively 2, 5 and 4; *m* of *x*_*a*2,*i*_^*ZG*88^, *x*_*d*1,*i*_^*ZG*88^, *x*_*d*2,*i*_^*ZG*88^ are respectively 2, 5 and 3; *m* of *x*_*a*2,*i*_^*G*3^, *x*_*d*1,*i*_^*G*3^, *x*_*d*2,*i*_^*G*3^ are respectively 2, 3 and 2 *m* of *x*_*a*2,*i*_^*T*1^, *x*_*d*1,*i*_^*T*1^, *x*_*d*2,*i*_^*T*1^ are respectively 2, 3 and 4;. The results of *PSR* are shown in Table 2. Then we can fit and predict landslide displacements using Voltterra filter and SVM models as:

3 $${x}_{i+1}=Volterra({X}_{i})\,{\rm{or}}\,{x}_{i+1}=SVM({X}_{i})$$

**Table 2:** *PSR* of each frequency component of ZG85 ~ ZG88, G3 and T1 cumulative displacements.

Displacements	Reconstructed phase spaces (input variables)	Output variables
ZG85	${X}_{a2,\,i}^{ZG85}=({x}_{a2,\,i}^{ZG85},{x}_{(a2,\,i-1)}^{ZG85}),i=2,3,\cdots ,113$	${x}_{a2,\,(i+1)}^{ZG85}$
	${X}_{d1,\,i}^{ZG85}=({x}_{d1,\,i}^{ZG85},\,{x}_{d1,\,(i-1)}^{ZG85},\,{x}_{d1,\,(i-2)}^{ZG85},\,{x}_{d1,\,(i-3)}^{ZG85}),i=4,5,\cdots ,113$	${x}_{d1,\,(i+1)}^{ZG85}$
	${X}_{d2,\,i}^{ZG85}=({x}_{d2,\,i}^{ZG85},\,{x}_{d2,\,(i-1)}^{ZG85},\,{x}_{d2,\,(i-2)}^{ZG85},\,{x}_{d2,\,(i-3)}^{ZG85}),i=4,5,\cdots ,113$	${x}_{d2,\,(i+1)}^{ZG85}$
ZG86	${X}_{a2,\,i}^{ZG86}=({x}_{a2,\,i}^{ZG86},{x}_{(a2,\,i-1)}^{ZG86}),i=2,3,\cdots ,113$	${x}_{a2,\,(i+1)}^{ZG86}$
	${X}_{d1,\,i}^{ZG86}=({x}_{d1,\,i}^{ZG86},\,{x}_{d1,\,(i-1)}^{ZG86},\,{x}_{d1,\,(i-2)}^{ZG86},\,{x}_{d1,\,(i-3)}^{ZG86},\,{x}_{d1,\,(i-4)}^{ZG86}),i=5,6,\cdots 113$	${x}_{d1,\,(i+1)}^{ZG86}$
	${X}_{d2,\,i}^{ZG86}=({x}_{d2,\,i}^{ZG86},\,{x}_{d2,\,(i-1)}^{ZG86},\,{x}_{d2,\,(i-2)}^{ZG86}),i=3,4,\cdots ,113$	${x}_{d2,\,(i+1)}^{ZG86}$
ZG87	${X}_{a2,\,i}^{ZG87}=({x}_{a2,\,i}^{ZG87},{x}_{(a2,\,i-1)}^{ZG87}),i=2,3,\cdots ,113$	${x}_{a2,\,(i+1)}^{ZG87}$
	${X}_{d1,\,i}^{ZG87}=({x}_{d1,\,i}^{ZG87},\,{x}_{d1,\,(i-1)}^{ZG87},\,{x}_{d1,\,(i-2)}^{ZG87},\,{x}_{d1,\,(i-3)}^{ZG87},\,{x}_{d1,\,(i-4)}^{ZG87}),i=5,6,\cdots 113$	${x}_{d1,\,(i+1)}^{ZG87}$
	${X}_{d2,\,i}^{ZG87}=({x}_{d2,\,i}^{ZG87},\,{x}_{d2,\,(i-1)}^{ZG87},\,{x}_{d2,\,(i-2)}^{ZG87},\,{x}_{d2,\,(i-3)}^{ZG87}),i=4,5,\cdots ,113$	${x}_{d2,\,(i+1)}^{ZG87}$
ZG88	${X}_{a2,\,i}^{ZG88}=({x}_{a2,\,i}^{ZG88},{x}_{(a2,\,i-1)}^{ZG88}),i=2,3,\cdots ,113$	${x}_{a2,\,(i+1)}^{ZG88}$
	${X}_{d1,\,i}^{ZG88}=({x}_{d1,\,i}^{ZG88},\,{x}_{d1,\,(i-1)}^{ZG88},\,{x}_{d1,\,(i-2)}^{ZG88},\,{x}_{d1,\,(i-3)}^{ZG88},\,{x}_{d1,\,(i-4)}^{ZG88}),i=5,6,\cdots 113$	${x}_{d1,\,(i+1)}^{ZG88}$
	${X}_{d2,\,i}^{ZG88}=({x}_{d2,\,i}^{ZG88},\,{x}_{d2,\,(i-1)}^{ZG88},\,{x}_{d2,\,(i-2)}^{ZG88}),i=3,4,\cdots ,113$	${x}_{d2,\,(i+1)}^{ZG88}$
G3	${X}_{a2,\,i}^{G3}=({x}_{a2,\,i}^{G3},{x}_{(a2,\,i-1)}^{G3}),i=2,3,\cdots ,56$	${x}_{a2,\,(i+1)}^{G3}$
	${X}_{d1,\,i}^{G3}=({x}_{d1,\,i}^{G3},\,{x}_{d1,\,(i-1)}^{G3},\,{x}_{d1,\,(i-2)}^{G3}),i=3,4,\cdots ,56$	${x}_{d1,\,(i+1)}^{G3}$
	${X}_{d2,\,i}^{G3}=({x}_{d2,\,i}^{G3},\,{x}_{d2,\,(i-1)}^{G3}),i=2,3,\cdots ,56$	${x}_{d2,\,(i+1)}^{G3}$
T1	${X}_{a2,\,i}^{T1}=({x}_{a2,\,i}^{T1},{x}_{(a2,\,i-1)}^{T1}),i=2,3,\cdots ,88$	${x}_{a2,\,(i+1)}^{T1}$
	${X}_{d1,\,i}^{T1}=({x}_{d1,\,i}^{T1},\,{x}_{d1,\,(i-1)}^{T1},\,{x}_{d1,\,(i-2)}^{T1}),i=3,5,\cdots ,88$	${x}_{d1,\,(i+1)}^{T1}$
	${X}_{d2,\,i}^{T1}=({x}_{d2,\,i}^{T1},\,{x}_{d2,\,(i-1)}^{T1},\,{x}_{d2,\,(i-2)}^{T1},\,{x}_{d2,\,(i-3)}^{T1}),i=4,5,\cdots ,88$	${x}_{d2,\,(i+1)}^{T1}$

### Displacements prediction using chaotic WA-Volterra and chaotic WA-SVM models

In order to train and test the Volterra filter and SVM models, the ZG85 ~ ZG88 landslide displacements are divided into two subsets: the former 94 months of ZG85 ~ ZG88 GPS monitoring landslide displacements from June 2003 to March 2011 are adopted to train the model, while the remaining later 20 landslide displacements are adopted to test the models. The same as the Shuping landslide, there are a total of 57 months of monitoring data on the Baijiabao landslide, the former 42 monthly displacement time series of G3 from December 2006 to May 2010 are used to train the models, while the remaining 15 data points from June 2010 to August 2011 are used to test the models. Meanwhile, a total of 89 months of displacements on the Xintan landslide were monitored, the former 74 monthly displacements of T1 from January 1978 to February 1984 are used to train the models, while the remaining 15 displacements are used to test the models. The input-output variables of the chaotic WA-Volterra and chaotic WA-SVM models are obtained as shown in Table 2.

**Figure 10:**
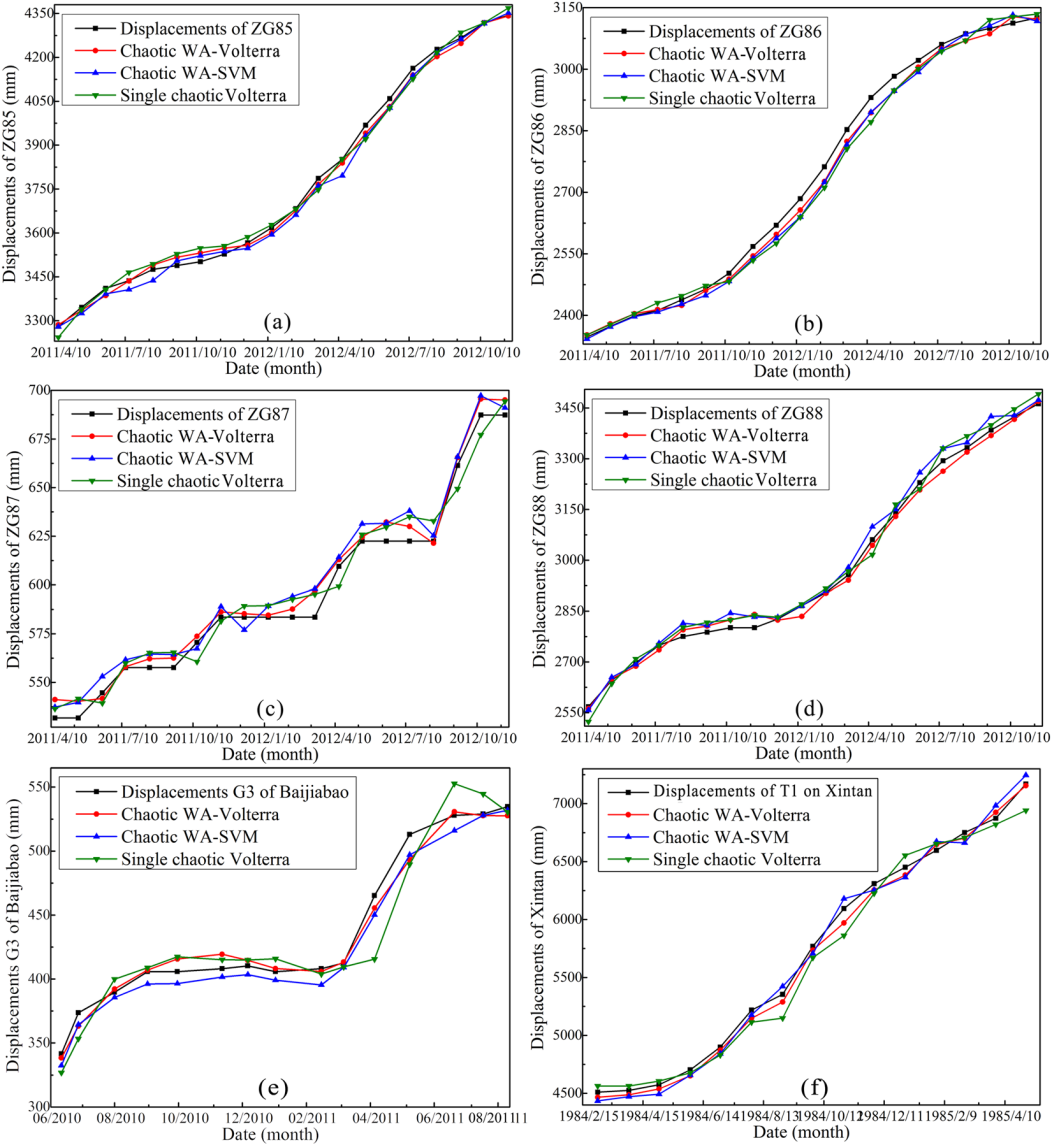
Comparison between predicting and monitoring the monthly ZG85 ~ ZG88 displacements of Shuping landslide, G3 displacements of Baijiabao landslide, and T1 displacements of Xintan landslide (drawn by Yuanyao Li).

The second-order Volterra filter model is used to construct the models for predicting the landslide displacements on Shuping landslide and Baijiabao landslide. The predictive results of the chaotic WA-Volterra model are shown in Fig. 10. To compare Volterra filter model with SVM model, the same input-output variables that have been used in the chaotic WA-Volterra model are used again in the chaotic WA-SVM model. The optimal parameter combination (*C*, *ε*, *γ*) of SVM is shown in Table 3. In addition, single chaotic Volterra filter model is also adopted in this study for comparison. The final predictive values of chaotic WA-SVM and single chaotic Volterra models are also shown in Fig. 10, and their prediction performances are assessed in Table 4.

**Table 3:** Parameters of the SVM model.

	GPS points	Frequency components	(*C*, *ε*, *γ*)
Chaotic WA-Volterra, Chaotic WA-SVM	ZG85	*a*2	(97.64, 0.005, 0.48)
		*d*1	(18.12, 0.01, 0.18)
		_*d*2_	(53.38, 0.005, 0.28)
	ZG86	*a*2	(486.52, 0.001, 0.31)
		*d*1	(45.42, 0.005, 0.19)
		*d*2	(38.22, 0.003, 0.32)
	ZG87	*a*2	(974.83, 0.005, 0.35)
		*d*1	(20.28, 0.008, 0.21)
		*d*2	(63.18, 0.031, 0.37)
	ZG88	*a*2	(52.47, 0.005, 0.39)
		*d*1	(42.36, 0.011, 0.21)
		*d*2	(73.62, 0.008, 0.22)
	G3	*a*2	(137, 0.02, 0.38)
		*d*1	(526, 0.06, 0.31)
		*d*2	(688, 0.005, 0.41)
	T1	*a*2	(73, 0.03, 0.45)
		*d*1	(81, 0.05, 0.33)
		*d*2	(125, 0.002, 0.32)

**Table 4:** Displacements prediction results of chaotic WA-Volterra, chaotic WA-SVM and single chaotic Volterra models for ZG85 ~ ZG88 of Shuping, G3 of Baijiabao and T1 of Xintan landslides.

GPS point	Prediction model	*RMSE*(*mm*)	*MAPE*(%)
ZG85	Chaotic WA-Volterra	19.13	0.45
	Chaotic WA-SVM	23.89	0.56
	Single chaotic Volterra	28.46	0.72
ZG86	Chaotic WA- Volterra	20.31	0.63
	Chaotic WA- SVM	24.22	0.76
	Single chaotic Volterra	29.67	0.83
ZG87	Chaotic WA- Volterra	6.32	0.86
	Chaotic WA- SVM	8.17	0.93
	Single chaotic Volterra	8.66	0.96
ZG88	Chaotic WA- Volterra	19.17	0.54
	Chaotic WA- SVM	23.64	0.66
	Single chaotic Volterra	26.48	0.75
G3	Chaotic WA- Volterra	7.88	1.59
	Chaotic WA- SVM	9.57	1.72
	Single chaotic Volterra	18.22	3.12
T1	Chaotic WA- Volterra	57.19	0.009
	Chaotic WA- SVM	72.93	0.012
	Single chaotic Volterra	116.92	0.016

## Discussion

### Chaos characteristics identify

The calculated *LLE* and correlation dimensions values show that there are chaos characteristics in the GPS monitoring landslide displacements. Hence, we are sure that the ZG85 ~ ZG88 GPS of Shuping landslide, G3 of Baijibao landslide and T1 cumulative displacements of Xintan landslide can be forecasted using the chaos-based models. The predictive results shown in Fig. 10 and Table 4 suggest that, the chaotic WA-Volterra, chaotic WA-SVM and single chaotic Volterra models accurately predict the GPS monitoring cumulative displacements as a whole. We can presume that appropriate input and output variables for the three nonlinear models are acquired from the reconstructed phase spaces, and can further extrapolate that the original nonlinear evaluation process of landslide displacement time series is effectively rebuilt by the *PSR* method of chaos theory.

### Comparison between volterra filter and svm models

The prediction performances of the three models are also shown in Fig. 10 and Table 4, which show that the chaotic WA-Volterra model is more accurate and credible than the chaotic WA-SVM model. We can conclude that the Volterra filter model is more appropriate for a finite nonlinear displacement time series than the SVM model. In actual, several nonlinear models are present to describe the nonlinear system, including Volterra filter, Taylor series and Wiener series, *et al*.^34^. Volterra filter model is one of the most popular nonlinear models for nonlinear dynamic time series prediction. There are several reasons, from a mathematical point of view, Voltera filter is essentially a functional series expansion of nonlinear time-invariant systems, and can also be regarded as the generalization of one-dimensional convolution in multidimensional convolution space^48^. From a physical point of view, the Volterra filter, which is similar to the linear impulse response function, can describe the substantive features of the nonlinear system with clear physical meaning. Moreover, the Volterra filter model can fit the nonlinear continuous functions with arbitrary precision because of its universal characteristic and ability of adaptive memory^49^. Similarly, the GPS monitoring landslide displacement time series is a nonlinear system with dynamic response and temporal memory features. Hence, the Volterra filter model can be used to accurately fit and predict the dynamic evaluation behavior of landslide displacements.

### Comparisons between chaotic WA-Volterra and single chaotic Volterra models

Figure 10 and Table 4 show that the predictive displacements of both chaotic WA based models deviate slightly from the real monitoring displacements, indicating that both chaotic WA based models have great prediction performances. On the contrary, results in Fig. 10 also show that, cumulative displacements of ZG85 ~ ZG88, G3 and T1 are underestimated overall by the single chaotic Volterra model. Specially, the chaotic Volterra model has difficulty in estimating the step-like features of these cumulative displacements, while chaotic WA-Volterra model overcomes this difficulty. In can be concluded that the chaotic WA based models have remarkably higher prediction performance than the single chaotic Volterra model. This is because that the nonlinear and non-stationary characteristics of GPS monitoring cumulative displacements are addressed reasonably well by the WA method before predict cumulative displacements using Volterra filter and SVM models. The “noises” existed in the original GPS monitoring landslide cumulative displacements are effectively removed by the WA method. As a result, the valuable information with less “noises” in the low- and high-frequency components can be fully extracted to train and test the Volterra filter and SVM models.

In addition to wavelet analysis, some other methods have also been proposed to implement time series decomposition and errors reduction. These methods include empirical mode decomposition^23^, principal component decomposition^50,51^, Fourier transformation^52^, polynomial decomposition^10^, *et al*. Comparing to the other time series decomposition methods, wavelet analysis is used more widely and is considered to be of higher decomposition efficiencies in both time and frequency domains^53,54^. Especially, Wickersham, Li and Lin^55^ shows that wavelet analysis has advantages of great applicability to discrete signals, insensitivity to the choice of wavelet basis, and insensitivity to the target signal extracted from the raw measurements comparing to traditional Fourier and PCA methods. Hence, this study selects wavelet analysis to decompose GPS monitoring landslide displacement time series for building landslide displacements prediction model.

Unfortunately, the present chaotic WA-Volterra model is just an offline modeling procedure without practical application. Hence, it is necessary to software this proposed model for online real-time landslide displacement prediction. In addition, we can still improve these three models. For example, it is necessary to obtain a longer length displacement time series of the displacement rather than a limit length landslide displacement time series. As a result, the reconstructed strange attractors can be fully unfolded to reflect the original strange attractors.

## Conclusions

In this study, chaos evidences of landslide displacements of Shuping landslide, Baijiabao landslide and Xintan landslide are determined based on LLE and *CD* methods, and a novel forecasting model is proposed for landslide displacements prediction. We can conclude that chaos characteristics existed in the nonlinear landslide displacements and these displacements are effectively predicted by the proposed chaotic WA-Volterra model. In addition, the chaotic WA-Volterra model obtains more accurate predictive displacements than the chaotic WA-SVM and single chaotic Volterra models. The main contributions of this study contain chaos characteristics identification of landslide displacements, landslide displacement time series decomposition using wavelet analysis, and Volterra filter model proposed for model construction.

## Methods

**Figure 11:**
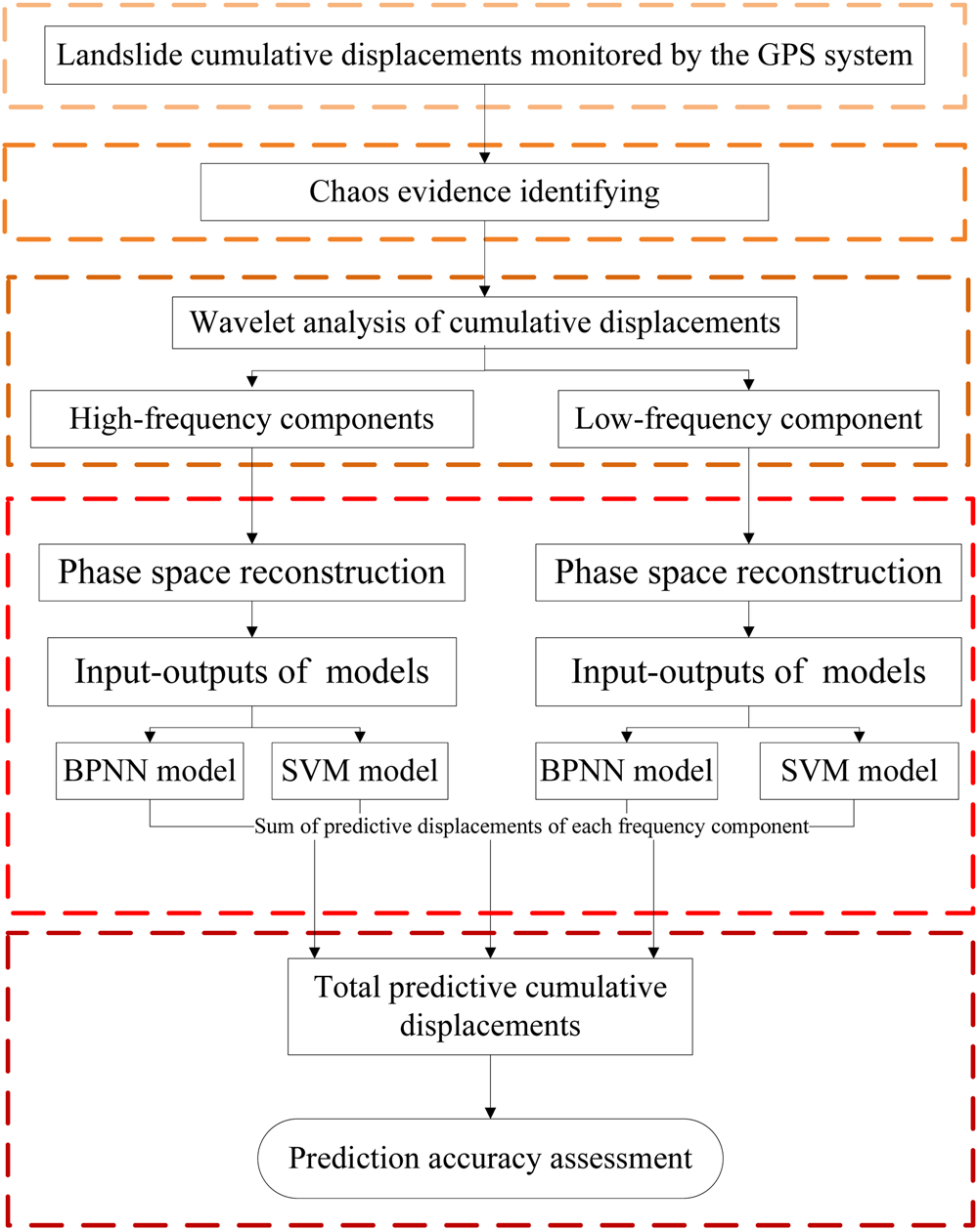
Flowchart of the chaotic WA-Volterra model (Drawn by Yuanyao Li).

The present chaotic WA-Volterra model has five steps as shown in Fig. 11: (1) the landslide cumulative displacements are obtained using the GPS system. (2) these cumulative displacement time series are normalized. (3) the chaos evidences of cumulative displacements are determined. (4) the cumulative displacements are decomposed into different low- and high-frequency components using WA method. (4) phase space of each frequency component is reconstructed using chaos theory, then Volterra filter model are trained and tested using these inputs-outputs obtained from reconstructed phase spaces. (5) through summing the predictive displacements of each frequency component, we can obtain the predictive cumulative displacements.

### Landslide displacement monitoring using the GPS system

#### Introduction to the GPS system

**Figure 12:**
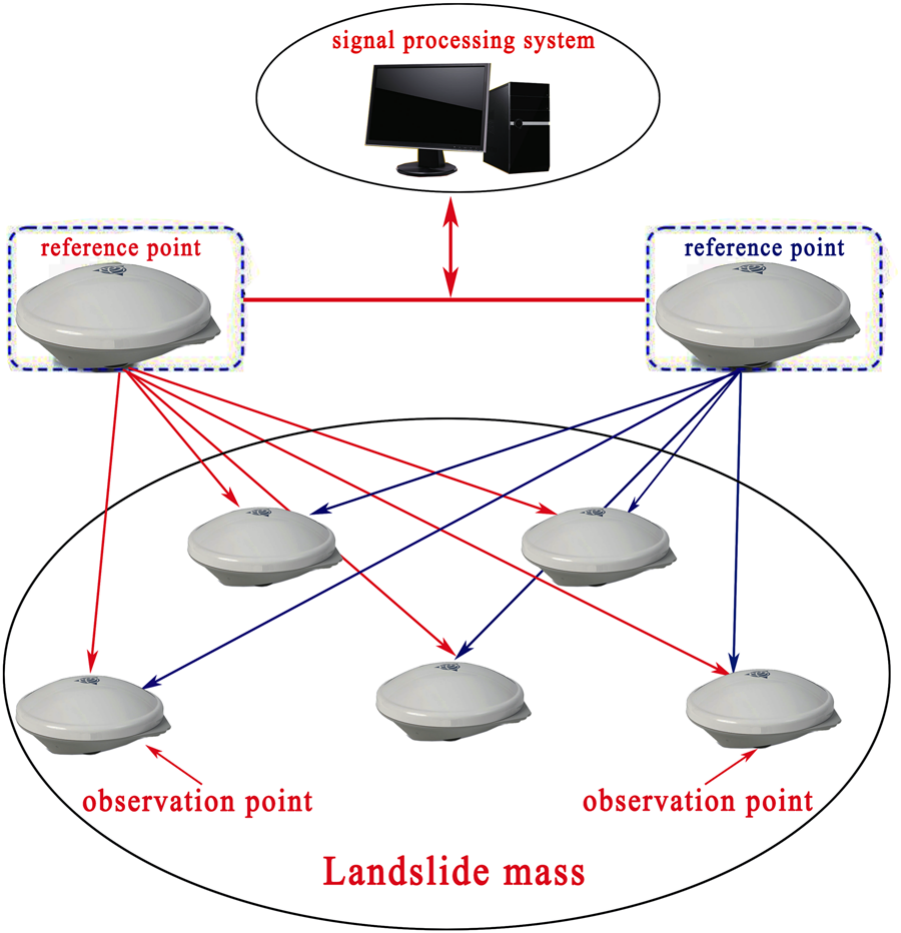
Structure of the GPS system (drawn by Yuanyao Li).

The proposed GPS system is composed of the signal receiver, signal processing and displacement presentation subsystems. The architecture of the GPS system is shown in Fig. 12. In the signal receiver subsystem, some GPS positioning methods used for monitoring the landslide displacements are proposed, such as the static relative positioning measurement^56^ and carrier phase measuring method^57^, *et al*. The static relative positioning measurement, which is of millimeter accuracy, is used in this study to construct the GPS monitoring system. The reason is that the static relative positioning measurement method can eliminate the orbit and atmosphere errors by a spatial correlation between the reference point and measuring points^25^. At the same time, the errors between the clocks in the GPS receivers and related satellites can also be removed by this method.

The production type of the GPS antenna is Trimble R8 GNSS with multi-channels and multi-frequencies. These GPS positioning messages contain a carrier phase, pseudo-range, the known coordinates and some other data^56^. The reference points and observation points receive one-day signals synchronously as the signal monitoring phase. The GPS antennas should receive at least five satellite signals at the same time, and then, the received signals will be transmitted to the signal processing subsystem.

The signal processing subsystem is mainly composed of GPS signal processing software (GAMIT/GLOBK software^41^) and a computer server, with display software. In the computer server, GAMIT/GLOBK software is used to obtain *mm* level positioning results of the measured points. First, the three-dimensional coordinate values of all of the points are measured. Then, the three-dimensional coordinate values of the measured points are compared to the reference point to determine the landslide displacements of the measured points.

#### Error analysis of the GPS system

It is important to analyze the errors of the GPS system. The displacement errors of the GPS system can be mainly divided into the system errors and random errors. Most of the system errors are removed when the received GPS signals are processed based on the principle of differential GPS; as a result, the random errors with a few system residual errors are the main type of errors of the GPS monitoring displacements^25^. The random errors reduce the accuracy of the fitting and prediction of the non-linear models. The errors of the GPS system on the Shuping and Baijiabao landslides have been calculated and shown that the horizontal errors and vertical errors in the monitoring landslide displacements are less than ±2.00 mm and ±5.00 mm, respectively^58^. Hence, the impact of the total errors on the prediction accuracy of the chaotic WA based model is limited^6^.

### Data normalization

It is necessary to perform data normalization to help the Volterra filter and SVM models converge to the optimal solution before model building. The original cumulative displacements are transformed into the desired range of [0, 1] as:

4 $${x}_{i}=\frac{{x}_{old,i}-{x}_{old,{\rm{\min }}}}{{x}_{old,{\rm{\max }}}-{x}_{old,{\rm{\min }}}}$$

where *x*_*old*,*i*_(*i* = 1, 2, ……, *N*) are the monitoring cumulative displacements, ${x}_{old,{\rm{\min }}}$ and ${x}_{old,{\rm{\max }}}$ are respectively their lower and upper bounds, *N* is the number of cumulative displacements. The predictive normalized displacement is back-transformed, so as to obtain final predictive displacement. The result is shown in Eq. (5), where *y*_*i*_ is the predictive normalized cumulative displacement.

5 $${\hat{y}}_{i}={y}_{i}\times ({x}_{old,{\rm{\max }}}-{x}_{old,{\rm{\min }}})+{x}_{old,{\rm{\min }}}$$

### Wavelet analysis

Wavelet analysis (WA)^21^ is a significant tool which deals with the time series based on the processes of dilation and translation. we define the mother wavelet function *φ*(*t*):

6 $${\int }_{-\infty }^{+\infty }\varphi (t)dt=0$$

7 $${\varphi }_{a,b}(t)={|a|}^{-\frac{1}{2}}\varphi (\frac{t-b}{a})$$

where *a* and *b* are set to real numbers, and *φ*_*a*,*b*_(*t*) donates the successive wavelet. This study use the discrete wavelet transform (DWT)^59^, one wavelet analysis method, to decompose the landslide displacement time series. The parameters *a* and *b* of DWT can be determined as:

8 $${\varphi }_{u,v}(\frac{t-b}{a})={{a}_{0}}^{-\frac{u}{2}}{\varphi }^{\ast }(\frac{t-n{b}_{0}{a}_{0}^{u}}{{a}_{0}^{u}})$$

where *a*_0_ is usually set to two, *u* and *v* are adopted to affect the processes of wavelet transform, *b*_0_ is usually set to one. Then a finite discrete time series *f*(*t*) can be defined by DWT as:

9 $${W}_{f}(u,v)={2}^{-\frac{u}{2}}\mathop{\sum }\limits_{t=0}^{K-1}f(t){\varphi }^{\ast }({2}^{-u}i-v)$$

where *W*_*f*_(*u*, *v*) is regarded as the wavelet coefficient, *a* = 2^*u*^, *b* = 2^*u*^*v* and *K* = 2^*U*^; *v* is regarded as time translation parameter varying between 0 and 2^*U*−*u*^ − 1, where 1 < *u* < *U*. The DWT can effectively decompose the cumulative displacement time series into the trend, period and fluctuation characteristics using its high-pass filters and low-pass filters. Meanwhile, the Mallat algorithm^60^ is used in the DWT method, then the cumulative displacement *x*_*i*_ is decomposed as:

10 $${x}_{i}={x}_{a,i}+{x}_{d1,i}+{x}_{d2,i}+\cdots \cdots +{x}_{dL,i}$$

where *L* donates the decomposition level; *x*_*a*,*i*_ presents the low-frequency component suggesting the trend characteristics; *x*_*d*1,*i*_, *x*_*d*2,*i*_, ……, *x*_*dL*,*i*_ respectively donates the first, second, ……, *L*th level high-frequency components reflecting the periodic, fluctuation characteristics of the cumulative displacement time series. In addition, The *db*4 is used in this study as wavelet function according to Li, Huang, Jiang, Huang and Chang^25^. Further, Eq. (11) is used to determine the number of decomposition levels according to Nourani, Alami and Aminfar^61^, where *N* presents the length of the GPS monitoring landslide displacement.

11 $$L=\,{\rm{int}}[\log (N)]$$

### Chaos theory and phase space reconstruction

#### Introduction of chaos theory

Chaos theory attempts to explain the fact that complex and unpredictable results can and will occur in nonlinear systems. A landslide displacement system has a temporal deterministic complexity. It has been proved by many studies that there is evidence of chaos in the monitoring displacement time series^6,28^. However, it is still necessary to determine the chaos evidence of the GPS monitoring landslide displacements of Shuping and Baijiabao landslides in this study. The reason is that the non-linearity of the landslide displacements can be established by determining the chaos evidence of the landslide displacements^6^. The chaos evidence of nonlinear time series is mainly identified through the Largest Lyapunov Exponent (LLE)^62^ and Correlation Dimension (*CD*) methods^63^. This study uses both two methods to determine the chaos characteristics of GPS monitoring landslide displacements.

#### Phase space reconstruction

According to PSR, for example, a displacement time series *x*_*i*_ can be fully embedded into *m*-dimensional phase space. As a result, the inputs and output of nonlinear predictor can be obtained from the reconstructed *m*-dimensional phase spaces^35^. The inputs of nonlinear predictor are represented by vector *X*_*i*_ as:

12 $${X}_{i}=({x}_{i},{x}_{i-\tau },{x}_{i-2\tau },\,\cdots \cdots \,,\,{x}_{i-(m-1)\tau })$$

where *i* = (*m* − 1)*τ* + 1,(*m* − 1)*τ* + 2, ……, *N* is the number of vectors, *N* is the number of displacements, *τ* is delay time and *m* is embedding dimension (*m* ≥ *d*, where *d* is the dimension of the attractor). It is important to select appropriate delay time and embedding dimension values to effectively reconstruct the original phase spaces of the GPS monitoring displacements. The delay time of displacement time series is generally set to one for landslide displacement predictions because the displacements are noisy and the length of displacement time series is very limit^24^. In addition, the false nearest neighbor (FNN) method^64^ is used to calculate the appropriate *m*, this is because FNN is insensitive to finite and noisy displacements.

### Identifying of chaos evidences of landslide cumulative displacements

#### Chaos evidence identification using LLE method

Small-data set method^65^ is used to calculate the *LLE* value of landslide displacement. The LLE is set to *L* in this study then the average divergence at time *t* can be defined as

13 $$d(t)=k{e}^{Lt}$$

where *k* is a constant that normalizes the initial separation. The reconstructed trajectory *X* can be expressed as:

14 $$X={({X}_{1},{X}_{2},\cdots \cdots ,{X}_{M})}^{T}$$

where *X*_*i*_ is the *i*th data point of the dynamic system, *M* is the number of data points on the reconstructed attractor, and each *X*_*i*_ is given by Eq. (12). The nea rest neighbor *X*_*j*_^*Near*^ is found by searching for the point that minimizes the distance to the particular reference point *X*_*j*_:

15 $${d}_{j}(0)=\mathop{{\rm{\min }}}\limits_{{X}_{j}^{Near}}\Vert {X}_{j}-{X}_{j}^{Near}\Vert $$

where *d*_*j*_(0) is the initial distance from the *j*th point to its nearest neighbor, and || || denotes the Euclidean norm. Based on the definition of *L* given in Eq. (13), the *j*th pair of nearest neighbors diverges approximately at the rate given by

16 $${d}_{j}({t}_{i})\approx {k}_{j}{e}^{L(i\Delta t)}$$

where *t*_*i*_ = *i*Δ*t*, Δ*t* is the sampling period of the time series, and *k*_*j*_ is the initial separation of the *j*th pair of nearest neighbors. Taking the logarithm of both sides of Eq. (16), we have

17 $$\mathrm{ln}\,{d}_{j}({t}_{i})\approx \,\mathrm{ln}\,{k}_{j}+L(i\Delta t)$$

Equation (18) represents a set of approximately parallel lines (for *j* = 1, 2, ……, *M*), each of which has a slope that is roughly proportional to *L*. The value of *L* can be calculated by

18 $$y({t}_{i})=\frac{1}{\Delta t}\langle \mathrm{ln}\,{d}_{j}({t}_{i})\rangle $$

where 〈·〉 denotes the average over all *j*. This process of averaging is one very important step to determine an accurate *L* based on a finite and noisy time series. Landslide displacement is considered as a chaotic time series when *LLE* value satisfies the condition for chaos, i.e., *LLE* > *0*.

#### Chaos evidence identification using *CD* method

*CD* method uses a non-integer fractal dimension to characterize the chaos characteristic of landslide displacement time series. In this study, the Grassberger-Procaccia (G-P) method^66^ is proposed to calculate the *CD* values of ZG85 ~ ZG88, G3 and T1 landslide displacements. Suppose two points *X*_*i*_ and *X*_*j*_ in the reconstructed phase space, we can describe *CD* as:

19 $$C(r)=\mathop{\mathrm{lim}}\limits_{N\to \infty }\frac{2}{M(M-1)}\mathop{\sum }\limits_{i=1}^{M}\mathop{\sum }\limits_{\begin{array}{c}j=1\\ j\ne i\end{array}}^{M}S(r-\Vert {X}_{i}-{X}_{j}\Vert )$$

where *S* is the Heaviside step function, the *S*(*u*) is set to 1 if *u* ≥ 0 and is set to 0 if *u* < 0; *r* represents the radius of the sphere centered on *X*_*i*_ or *X*_*j*_. Suppose the *D*(*m*) as *CD* value, *C*(*r*) can be *r*elated to radius *r* as Eq. (20) when the nonlinear landslide displacement is featured by an attractor.

20 $$C(r)\propto {r}^{D(m)}$$

Take the logarithm of Eq. (20), then we can rearrange it as:

21 $$D(m)=\mathop{\mathrm{lim}}\limits_{r\to 0}\frac{\mathrm{ln}\,C(r)}{\mathrm{ln}\,r}$$

Based on Eq. (21), we can obtain a series of *D*(*m*) through the increase of *m* value. When the nonlinear landslide displacements are of chaos characteristic, the *D*(*m*) will continuously increases and then converges to a constant with the increase of *m* value^67^.

### Volterra filter model

The Volterra filter is a model which can be adopted to predict the non-linear time series^68^. Volterra filter model has the ability to obtain the memory information through the model training and testing processes. We can get a second-order Volterra filter model when setting the degree of Volterra filter as 2. Relational literature shows that a second-order Volterra filter model has efficient performance for nonlinear time series prediction:

22 $${\rm{y}}({\rm{t}})=\{\begin{array}{c}\mathop{\sum }\limits_{{\rm{n}}=1}^{{\rm{S}}}\mathop{\sum }\limits_{{\tau }_{1}=1}^{p}{{h}_{1}}^{(n)}(\tau ){x}_{n}(t-\tau )\\ +\mathop{\sum }\limits_{n=1}^{S}\mathop{\sum }\limits_{{\tau }_{1}=1}^{p}\mathop{\sum }\limits_{{\tau }_{2}=1}^{p}{{h}_{2s}}^{(n)}({\tau }_{1},{\tau }_{2}){x}_{n}(t-{\tau }_{1}){x}_{n}(t-{\tau }_{2})\\ +\mathop{\sum }\limits_{{n}_{1}=1}^{S}\mathop{\sum }\limits_{{n}_{2}=1}^{{n}_{1}-1}\mathop{\sum }\limits_{{\tau }_{1}=1}^{p}\mathop{\sum }\limits_{{\tau }_{2}=1}^{p}{{h}_{2x}}^{({n}_{1},{n}_{2})}({\tau }_{1},{\tau }_{2}){x}_{n1}(t-{\tau }_{1}){x}_{n2}(t-{\tau }_{2})+{{\rm{\xi }}}_{{\rm{t}}}\end{array}$$

where *y*(*t*) is single output prediction value, *x*_1_, *x*_2_, *x*_3_$\cdots$*x*_*S*_ are time series and they are considered as input variable values, *S* represents the number of input variables, *N* donates length of time series about input variables, *p* represents the memory value which showing a significant lag relationship between different input variables, *ξ*_*t*_ represents the noises produced in model building.

Equation (22) shows that the second-order cross-kernels *h*_2*x*_^(*n*^_1_^,*n*^_2_^)^ represents a second-order nonlinear interactive relationships between each unique pair of input variables named *x*_*n*1_ and *x*_*n*2_.We can simply Eq. (22) through combining the last two terms to yield Eq. (23) as:

23 $$\begin{array}{rcl}y(t) & = & \{\mathop{\sum }\limits_{n=1}^{S}\mathop{\sum }\limits_{\tau =1}^{p}{{h}_{1}}^{(n)}(\tau ){x}_{n}(t-\tau )\\  &  & +\,\mathop{\sum }\limits_{{n}_{1}=1}^{S}\mathop{\sum }\limits_{{n}_{2}=1}^{S}\mathop{\sum }\limits_{{\tau }_{1}=1}^{p}\mathop{\sum }\limits_{{\tau }_{2}=1}^{p}{{h}_{2x}}^{({n}_{1},{n}_{2})}({\tau }_{1},{\tau }_{2}){x}_{n1}(t-{\tau }_{1}){x}_{n2}(t-{\tau }_{2})+{{\rm{\xi }}}_{{\rm{t}}}\}\end{array}$$

The Orthogonal Least Squares-Error Reduction Ration^69^ is used in this study to estimate the relative parameters of two-order Volterra filter model. Finally, the proposed Volterra filter model can be adopted to predict each frequency component of landslide displacement time series.

### Support Vector machine

SVM^70^ is a time series prediction technique with good performance. we can approximate the regression function as follows:

24 $$F(X)=\omega \cdot \phi (X)+b$$

where *b* is a scalar threshold, *ω* is the weight vector, *ϕ*(*X*) is a high-dimensional feature space, and *ϕ*(*X*) is from the input space *X* by nonlinear mapping. The SVM model performs linear regression in the high-dimensional feature space by *ε*-insensitive loss. Then, we can estimate the coefficients *ω* and *b* by minimizing the regularized risk function Eq. (25)^71^:

25 $$\begin{array}{c}Min\,\frac{{\Vert \omega \Vert }^{2}}{2}\\ s.t.\,\{\begin{array}{c}{y}_{i}-\phi (\omega ,{x}_{i})-b\le \varepsilon \\ \phi (\omega ,{x}_{i})+b-{y}_{i}\le \varepsilon \end{array}\end{array}$$

Then, the regression problem can be transformed into the constrained formation:

26 $$\begin{array}{c}Min\,\frac{{\Vert \omega \Vert }^{2}}{2}+C\mathop{\sum }\limits_{i=1}^{n}({\zeta }_{i}+{\zeta }_{i}^{\ast })\\ s.t.\,\{\begin{array}{c}{y}_{i}-\langle \omega ,{x}_{i}\rangle -b\le \varepsilon +{\zeta }_{i}\cdot {\zeta }_{i}^{\ast }\ge 0\\ \langle \omega ,{x}_{i}\rangle +b-{y}_{i}\le \varepsilon +{\zeta }_{i}\cdot {\zeta }_{i}^{\ast }\ge 0\end{array}\end{array}$$

where the constant *C* stands for the penalty degree of the sample with an error that exceeds *e*. We can use the optimization method to maximize the function to deal with the dual problem as:

27 $$\begin{array}{c}\,Max\,\mathop{\sum }\limits_{i=1}^{n}{y}_{i}({a}_{i}+{a}_{i}^{\ast })-\varepsilon \,\mathop{\sum }\limits_{i=1}^{n}({a}_{i}+{a}_{i}^{\ast })\\ \,-\frac{1}{2}\mathop{\sum }\limits_{i=1}^{n}\mathop{\sum }\limits_{j=1}^{n}({a}_{i}-{a}_{i}^{\ast })({a}_{j}+{a}_{j}^{\ast })K({X}_{j}+{X}_{j})\\ s.t.\,\mathop{\sum }\limits_{i=1}^{n}({a}_{i}-{a}_{i}^{\ast })=0\,and\,0\le {a}_{i}\,,\,{a}_{i}^{\ast }\le C\end{array}$$

where *a*_*i*_ and *a*_i_^*^ are the Lagrange multiplier. The SVM predictor for the function fitting obtained by using the above-mentioned maximization function is then given as follows:

28 $$F(X)=\mathop{\sum }\limits_{i=1}^{n}({a}_{i}-{a}_{i}^{\ast })K({x}_{i},x)+b$$

In Eq. (28), those sample points are called as support vectors. The radial basis kernel function $K({x}_{i},x)=\phi ({X}_{i}\,,{X}_{j})$ is used as the kernel function of SVM^35^. Then particle swarm optimization (PSO) method is introduced to determine the appropriate parameters (*C*, *ε* and *r*) of SVM^35^.

### Accuracy assessment

In this study, we use two assessment methods, Root Mean Square Error (*RMSE*) and Mean Absolute Percentage Error (*MAPE*), to evaluate the prediction accuracies of each model^72^. The *RMSE* is calculated as

29 $$RMSE=\sqrt{\frac{\mathop{\sum }\limits_{i=1}^{{N}_{0}}{({x}_{old,i}-{\hat{y}}_{i})}^{2}}{{N}_{0}}}$$

where ${x}_{old,i}$ is the original cumulative displacement time sereis, ${\mathop{y}\limits^{\frown {}}}_{i}$ is the final predicted values, and *N*_0_ is the length of the predicted data. *MAPE* can be described as:

30 $$MAPE=\frac{1}{{N}_{0}}\mathop{\sum }\limits_{i=1}^{{N}_{0}}\frac{|{x}_{old,i}-{\hat{y}}_{i}|}{{x}_{old,i}}\times 100 \% $$

## Data Availability

The datasets generated during and/or analysed during the current study are available from the corresponding author on reasonable request.
